# Gene mutation analysis in 12 Chinese children with congenital nephrotic syndrome

**DOI:** 10.1186/s12882-018-1184-y

**Published:** 2018-12-29

**Authors:** Guo-min Li, Qi Cao, Qian Shen, Li Sun, Yi-hui Zhai, Hai-mei Liu, Yu An, Hong Xu

**Affiliations:** 1 Children’s Hospital of Fudan University, 399 Wanyuanlu, Shanghai, 201102 China; 2 Institutes of Biomedical Sciences of Fudan University, 220 Handanlu, Shanghai, 200433 China

**Keywords:** Chinese children, Congenital nephritic syndrome, *NPHS1* gene, *WT1* gene, *COQ6* gene

## Abstract

**Background:**

Congenital nephrotic syndrome (CNS) is characterised by increased proteinuria, hypoproteinemia, and edema beginning in the first 3 months of life. Recently, molecular genetic studies have identified several genes involved in the pathogenesis of CNS. A systematic investigation of the genes for CNS in China has never been performed; therefore, we conducted a mutational analysis in 12 children with CNS,with the children coming from 10 provinces and autonomous regions in China.

**Methods:**

Twelve children with CNS were enrolled from 2009 to 2016. A mutational analysis was performed in six children by Sanger sequencing in eight genes (*NPHS1*, *NPHS2*, *PLCE1*, *WT1*, *LAMB2*, *LMXIB*, *COQ6* and *COQ2*) before 2014, and whole-exome sequencing was used from 2014 to 2016 in another six children. Significant variants that were detected by next generation sequencing were confirmed by conventional Sanger sequencing in the patients’ families.

**Results:**

Of the 12 children, eight patients had a compound heterozygous *NPHS1* mutation, one patient had a de novo mutation in the *WT1* gene, and another patient with extrarenal symptoms had a homozygous mutation in the *COQ6* gene. No mutations were detected in genes *NPHS2*, *PLCE1*, *LAMB2*, *LMXIB*, and *COQ2* in the 12 patients.

**Conclusions:**

This study demonstrates that the majority of CNS cases (67%, 8/12 patients) are caused by genetic defects, and the *NPHS1* mutation is the most common cause of CNS in Chinese patients. A mutational analysis of *NPHS1* should be recommended in Chinese patients with CNS in all exons of *NPHS1* and in the intron-exon boundaries.

## Background

Congenital nephrotic syndrome (CNS) is a rare kidney disorder characterised by a high degree of proteinuria, hypoproteinemia, and edema occurring within 3 months after birth [1]. Based on its aetiology, CNS can be divided into primary and secondary disease [2]. The secondary disease is mainly caused by various pathogen infections, such as toxoplasma, syphilis, rubella virus, cytomegalovirus, and herpes simplex virus [1, 2]. The majority of patients with CNS show mutations in genes encoding key podocyte proteins that constitute the slit diaphragm (*NPHS1* and *NPHS2*); others are expressed in the podocyte membrane *(PLCE1*), mitochondria, (*COQ6*, *COQ2*) or glomerular basement membrane *(LAMB2*), and other genes encode transcription factors that are necessary for normal development (*WT1*, *LMX1B*) [2–11]. *NPHS1* and *NPHS2* mutations are the main causes of CNS, but *LAMB2* and *WT1* mutations have been detected in a few CNS patients in Europe and America [3, 12]. The *NPHS1* mutation is also a major cause of CNS in Japan, *but NPHS2* mutations have been found in few CNS patients, and no *WT1* mutations have been identified in patients with CNS [13]. A study from South Korea demonstrated that *NPHS1* and *WT1* mutations are the main causes of CNS and that *NPHS2* and *LAMB2* mutations are only found in a few patients [14]. In China, mutations of *NPHS1*, *WT1*, and *LAMB2* were identified in sporadic patients with CNS [15–18], and no other gene mutations were reported in patients with CNS. Case studies involving mutational analyses of *NPHS1* and *LAMB2* are limited, and all of these investigations are case reports [15–17]; in addition, an analysis of a single *WT1* mutation was performed in patients with steroid-resistant nephrotic syndrome (SRNS) [18], so it is not clear which gene is the major cause of CNS. In this study, we performed a systematic investigation of genes in 12 children with CNS from 12 unrelated families and from 10 provinces and autonomous regions in China.

## Methods

### Patients

In total, 12 Chinese CNS patients from 12 unrelated families were enrolled in this study. All patients had the typical clinical findings of both proteinuria and nephrotic syndrome onset between birth and 3 months of age, and there was no evidence of congenital infections. Screenings of the patients’ blood sera excluded the presence of antibodies for toxoplasma, syphilis, rubella virus, cytomegalovirus, herpes simplex virus, human immunodeficiency virus (HIV), and *Chlamydia trachomatis*. All cases were admitted to or followed up at our centre (Children’s Hospital of Fudan University) between 2009 and 2016. The study was approved by the Ethics Committee at the Children’s Hospital of Fudan University, Shanghai, China. All of the patients’ parents provided written informed consents.

### DNA sequencing

The genomic DNA of all patients and their parents was extracted and purified from peripheral leukocytes in whole-blood samples by a DNA isolation kit (Qiagen, Hilden, Germany).

### Sanger sequencing

Sanger sequencing was performed in six children before 2014. All exons of *NPHS1*, *NPHS2*, *PLCE1*, *LAMB2*, *LMXIB, COQ6*, and *COQ2*, as well as exons 8 and 9 of *WT1*, were amplified by the polymerase chain reaction (PCR) method. The primers for *NPHS1*, *NPHS2*, *PLCE1*, *WT1*, *LAMB2*, *LMXIB*, *COQ6*, and *COQ2* were designed on the basis of previously published information regarding the intron-exon boundaries [3, 5–8, 19, 20]. The PCR products were purified with a QIA Quick PCR Purification Kit (Qiagen, Hilden, Germany). The purified products were cycle-sequenced with Big Dye terminators (Applied Biosystems, Foster City, CA, USA), and the cycle sequence products were analysed with an automated sequencer (ABI Prism 310 Genetic Analyser). The novel mutations were investigated in a number of mutation databases on human populations, such as the ExAC Browser (http://exac.broadinstitute.org/), the 1000 Genomes Project (http://www.internationalgenome.org/), HGMD (http://www.hgmd.cf.ac.uk/ac/index.php), *and*, in the 100 healthy control subjects, by direct sequencing.

### Next generation sequencing

Whole-exome sequencing was performed in six children with CNS from 2014 to 2016. Targeted exome capture was performed on the genomic DNA from each subject by using the SureSelect Human All Exon Target Enrichment System (Agilent). The captured whole exomes were sequenced on the Illumina HiSeq 2500 Sequencer platform (Illumina, San Diego, CA, USA). A mean exome coverage of 92.68× was obtained to accurately call variants at 99.51% of the targeted exome. The read alignment to the human genome assembly hg19 was obtained by using the novoalign alignment tool (Novocraft Technologies). Variant callings and annotations were obtained using the Samtools mpileup utility and Annovar tools, respectively. To obtain the important candidate genes, the resulting variants were systematically annotated and filtered. The program ANNOVAR (http://www.openbioinformatics.org/annovar/) was used to annotate the variants of the information from various genetic variation databases**.** Based on the reported variant frequencies, the common variants were first excluded with a minor allele frequency (MAF) greater than 0.01, as represented in the 1000 Genomes Project. According to the variant locations within genes, the variants in the coding regions were given a higher priority, and the variants that altered the coding sequences (nonsynonymous) were selected. The deleteriousness of the selected variants was subsequently predicted by various bioinformatics programs (SIFT, Polyphen2, and NetGene2) (http://genetics.bwh.harvard.edu/pph2/index.shtml; http://sift.jcvi.org/; http://www.cbs.dtu.dk/services/NetGene2), and the variants were retained if their changes to the resulting proteins were damaging.

## Results

### Clinical data

**Fig. 1:**
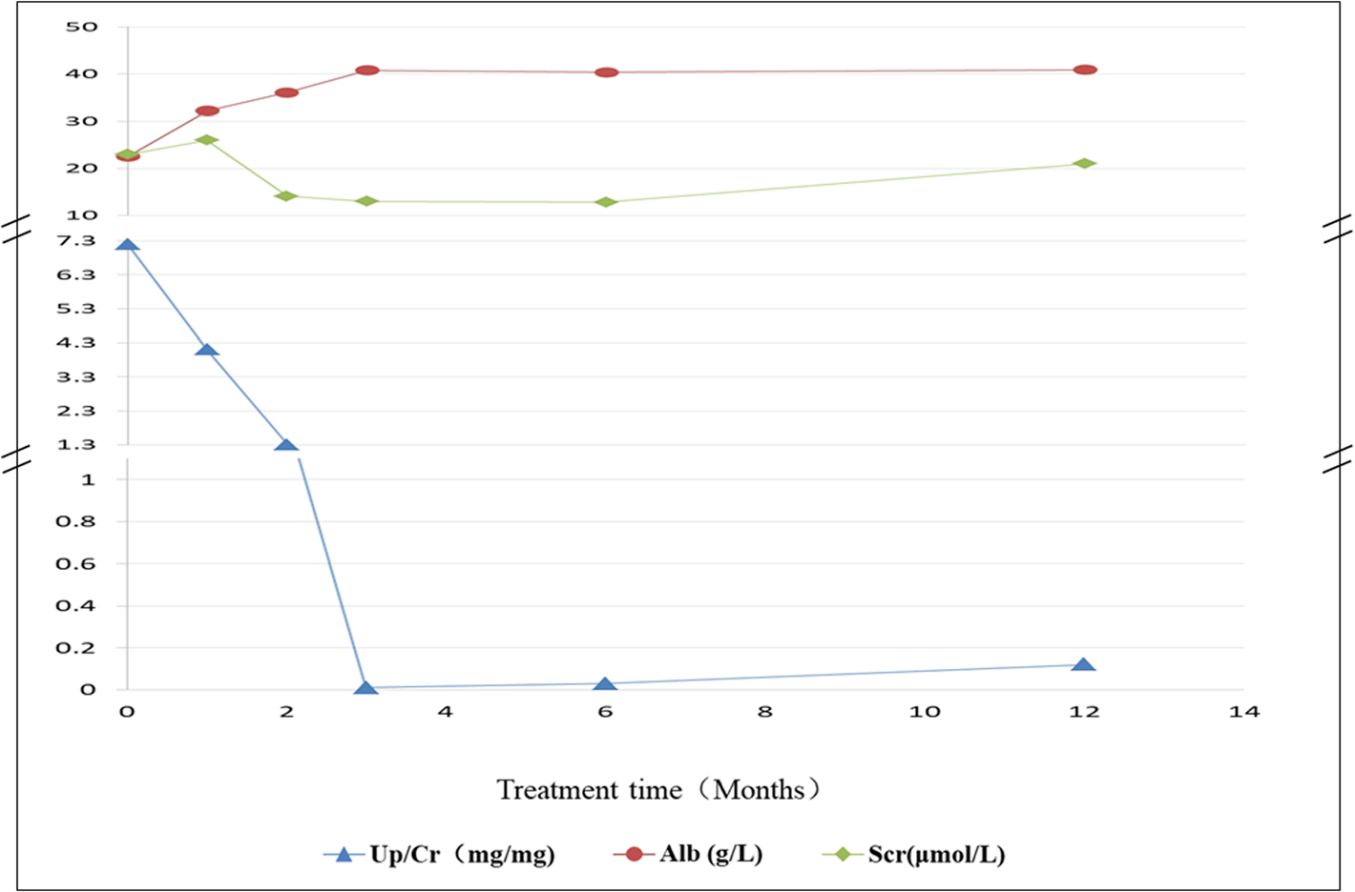
Change in Up/Cr, serum albumin, and creatinine after treatment with CoQ10

All of the 12 children were initially admitted to our centre due to a high degree of proteinuria and oedema. A renal biopsy was not performed in all children. Of these patients, 9 presented with heavy proteinuria and oedema during the first month of life, and 3 presented with heavy proteinuria and oedema during the first 2 months of life. Six children were male, and six children were female. Only nine children were followed up for 2–3 months, and their proteinuria did not resolve during the follow-up periods. They finally abandoned treatment due to economic reasons *and died from complication*s of infection. Three children (cases 5, 11, and 12) are being followed up in our centre. None of the patients received glucocorticoid therapy, except case 5, who did not respond to therapy. Case 5 was first diagnosed in 2006, and she was followed up at our centre. This child progressed to end-stage renal disease (ESRD) in 2009, after which she received peritoneal dialysis. She was switched to haemodialysis in 2013, due to a loss of peritoneum function, and received a renal transplantation in 2017. Case 11 presented with oedema and proteinuria after his birth. He progressed to end-stage renal disease at the age of nine months and received peritoneal dialysis since that time. A karyotype analysis revealed that he had 46, XY, which is a disorder of sex differentiation. Neither a Wilms tumour or a gonadoblastoma was found during his 3 year follow-up period. Case 12 was a premature infant. A cardiac murmur was found after his birth and continued to be present during his follow-up period. He presented with mild proteinuria at the age of two months. Due to oedema and motor retardation, he was admitted to our hospital for a further assessment at the age of 10 months. Upon admission, a clinical examination revealed that he could not sit, crawl, or even stabilise his head in an upright position. His height was at the third percentile, and his body weight was less than the fifth percentile. Laboratory tests revealed incidences of proteinuria (3+, total urine protein of 3.28 g/m^2^/24 h) and hypoalbuminemia (22.5 g/L). An ultrasound showed a pulmonary artery stenosis, a patent ductus arteriosus, an atrial septal defect, an ascending aorta velocity, and a persistent left superior vena cava. He was diagnosed with CNS, congenital heart disease, and motor retardation. At the age of 12 months, she received an oral administration of coenzyme Q10 after a genetic diagnosis. Her proteinuria gradually decreased and was completely in remission (Up/Cr 0.01 mg/mg) after three months of coenzyme Q10 therapy (see Fig. 1). Her motor retardation was also improved by coenzyme Q10 therapy. The clinical features of all patients included in the study are listed in Table 1.

**Table 1:** Clinical features of 12 children with CNS

Case	Age onset	Birth history				Weight of placenta	First symptom	Proteinuria (g/m^2^/24 h)	Serum albumin (g/l)	Age to ESRD	FH	ES
		Parity	GA(w)	BW(g)	Delivery							
1	At birth	G1P1	32	2400	C-sect	> 25% BW	Oedema	6.87	12.30	NA	No	No
2	13 D	G1P1	33	2450	C-sect	Normal	Oedema	8.74	8.26	NA	No	No
3	3 D	G1P1	37^+ 2^	2700	C-sect	Normal	Oedema	8.28	9.85	NA	No	No
4	1.5 M	G1P1	38	NA	NA	Normal	Oedema	6.32	12.8	NA	No	No
5	At birth	G2P1	37^+ 4^	3210	C-sect	Normal	Oedema	7.96	8.92	3 y	No	No
6	At birth	G2P1	NA	2760	N-labor	Normal	Oedema	7.58	10.20	NA	No	No
7	At birth	G1P1	38^+ 4^	3100	C-sect	Normal	Oedema	6.92	10.50	NA	No	No
8	15 D	G1P1	39	2850	C-sect	Normal	Oedema	2.88	28.20	NA	No	No
9	At birth	G1P1	38^+ 5^	3100	C-sect	Normal	Oedema	8.63	9.65	NA	No	No
10	1 M	G2P1	37^+ 2^	2880	N-labor	Normal	Oedema	7.89	11.20	NA	No	No
11	At birth	G2P1	36	2700	C-sect	Normal	Oedema	6.28	20.1	10 m	No	No
12	2 M	G1P1	38	1700	N-labor	Normal	Oedema	3.28	22.5	CKD-1	No	Yes

*NA* not available, *BW* Birthweight, *GA* Gestational age, *PW* Placental weight, *C-sect* Caesarean section, *N-labour* natural labour, *FH* Family history, *ES* Extrarenal symptom, *CKD-1* Chronic kidney disease stage 1,

### Sanger sequencing

The pathogenic mutations of *NPHS2*, *PLCE1*, *WT1*, *LAMB2*, *LMXIB*, *COQ6*, and *COQ2* were not found in the 6 children with CNS, but all of the subjects had double heterozygous mutations of *NPHS1*, as determined by Sanger sequencing (Table 2). A mutational analysis, via Sanger sequencing of the *NPHS1* gene in six of the patients’ families, showed that all of the mutations were from their parents, respectively.

**Table 2:** Genotypes of 10 children with CNS

Patient	Exon	Nucleotide change	amino acid substitution	Mutation type	Mutation status	Mutation origin	Novel mutation	Functional effect	
								PolyPhen	SIFT
*NPHS1* gene (RS: NM_004646.3)									
1	7	c.802C > T	p.R268X	Nonsense	Het	F	No	–	–
	10	c.1528A > C	p.C528G	Missense	Het	M	No	damaging	deleterious
2	20	c.2788C > T	p.Q930X	Nonsense	Het	F	Yes	–	–
	27	c.3442delC	p.Q1148fsX1179	Frameshift	Het	M	Yes	–	–
3	26	c.3325C > T	p.R1109X	Nonsense	Het	M	No	–	–
	27	c.3478C > T	p.A1160X	Nonsense	Het	F	No	–	–
4	13	IVS12 + 1C > A	NC	Splicing	Het	M	Yes	–	–
	24	c.3213delG	p.G1071fsX1142	Frameshift	Het	F	Yes	–	–
5	26	c.3325C > T	p.R1109X	Nonsense	Het	M	No	–	–
	27	IVS26DS-2A > T	NC	Splicing	Het	F	Yes	–	–
6	8	c.928G > A	p.D310N	Missense	Het	F	No	damaging	deleterious
	16	c.2131C > G	p.R711G	Missense	Het	M	No	damaging	deleterious
7	3	c.349G>A	p.E117K	Missense	Het	M	No	damaging	deleterious
	8	c.928G > A	p.D310N	Missense	Het	F	No	damaging	deleterious
9	10	c.1550G > T	p.T517K	Missense	Het	F	Yes	damaging	deleterious
	10	c.1559G > A	p.S520 L	Missense	Het	M	Yes	damaging	deleterious
*WT1* gene (RS: NM_024426.4)									
11	8	c.1334C > T	p.R445Q	Missense	Het	De novo	Yes	damaging	deleterious
*COQ6* gene (RS: NM_182476.2)									
12	9	c.1078C > T	p.R360W	Missense	Hom	Parents	Yes	damaging	deleterious

*NC* no change; −: No need for prediction; *RS* reference sequence

### Next generation sequencing

**Fig. 2:**
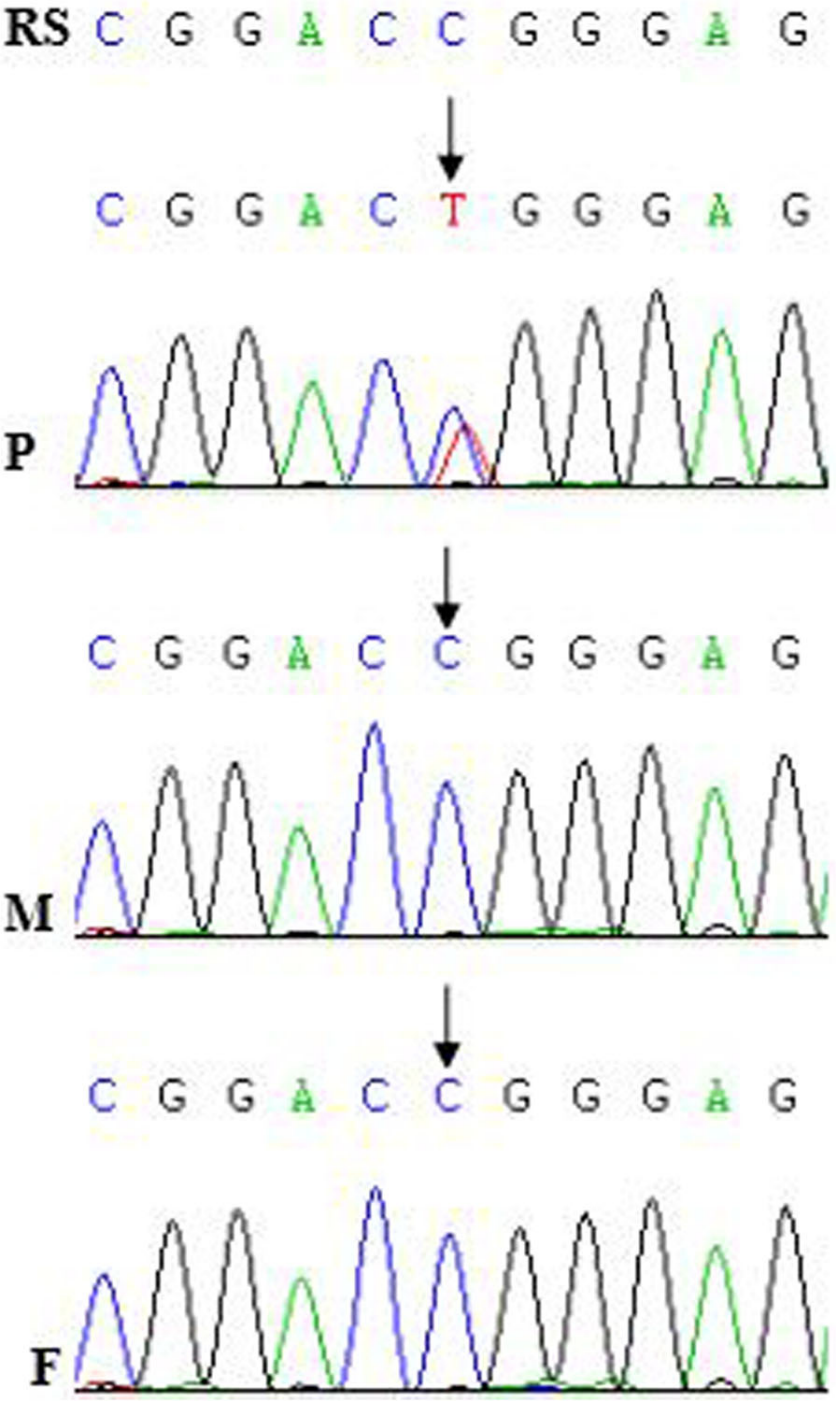
Mutational analysis in the *WT1* gene in the family of case 11. The sword showed a mutation. P: patient; F: father; M: mother

**Fig. 3:**
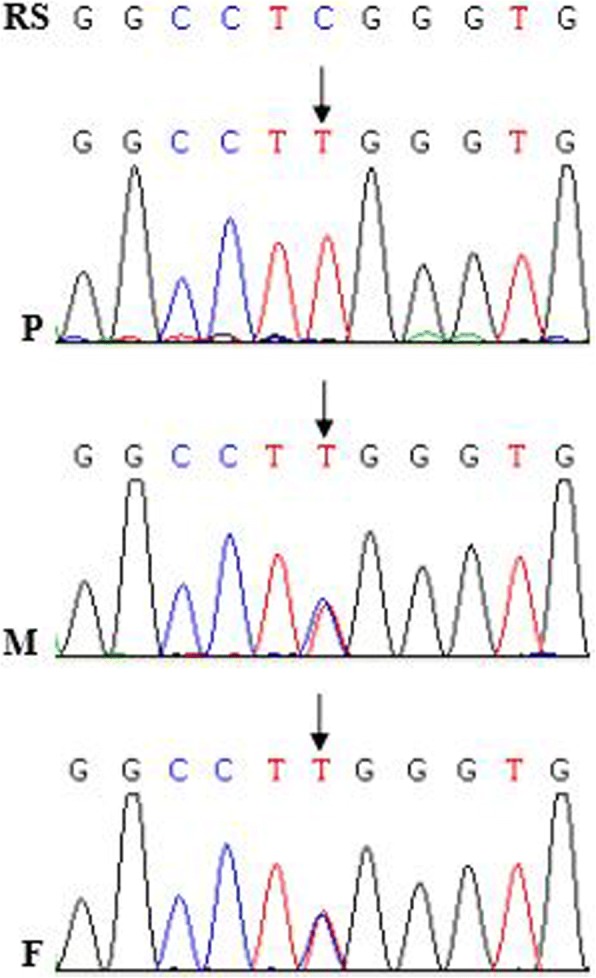
Mutational analysis in the *COQ6* gene in the family of case 12. The sword showed a mutation. P: patient; F: father; M: mother

Next generation sequencing was performed in the other six children. Two children had compound heterozygous mutations in the NPHS1 gene. Two children had a homozygous mutation in the *COQ6* gene and a heterozygous mutation in *WT1* gene, respectively (Table 2). No mutations were found in another two children. Mutations in the *NPHS1* and *COQ6* genes were confirmed by Sanger sequencing. Mutational analyses in those cases’ families showed that a c.1334C > T mutation in the WT1 gene was de novo (Fig. 2) and that other mutations in the *NPHS1* and *COQ6* genes were from their parents (Table 2 and Fig. 3), respectively.

### Functional prediction

The missense mutations p.D310N and p.R711G were assessed for their potential to disrupt protein function via SIFT and PolyPhen-2, and the predicted results are summarised in Table 2. NetGene2 predicted that a splicing mutation, IVS26DS-2A > T, affected the canonical site for splicing.

## Discussion

The frequency of the *NPHS1* gene mutation varies among different ethnic groups. In 98% of children with CNS in Finland, the disease is caused by *NPHS1* mutations [21]. Lenkkeri et al. reported that 80% (28/35) of CNS patients from North America, Europe, and North Africa have been identified to harbour mutations in the *NPHS1* gene [3]. A study from Europe showed that 84.8% (39/46) of paediatric CNS cases were explained by mutations in four genes. The distribution among these four genes was as follows: *NPHS1*, 39.8%; *NPHS2*, 39.8%; *WT1*, 2.2%; and *LAMB2*, 4.4% [12]. Another study from Europe revealed that 100% (15/15) of paediatric CNS cases were caused by mutations in the *NPHS1*, *NPHS2*, and *WT1* genes, with frequencies of 80, 7, and 13%, respectively [22]. Furthermore, no mutations of *PLCE1* and *LAMB2* have been detected [22]. Therefore, *NPHS1* is the main gene that causes CNS in Europe and North America. In 2005, a Japanese study showed that 4 out of 15 patients had *NPHS1* gene mutations (2 homozygous, 2 heterozygous), 2 patients had *NPHS2* gene mutations (1 homozygous, 1 heterozygous), and no patients had *WT1* gene mutations [13]. In 2009, another Japanese study reported that 5 out of 5 patients had *NPHS1* gene mutations, and the authors considered *NPHS1* to be the main causative gene of CNS in Japan [23]. A study from North Korea demonstrated that *NPHS1* and *WT1 g*ene mutations were the major causes of CNS and that *NPHS2* and *LAMB2* gene mutations were found in only a few CNS patients [14]. An *NPHS1* mutation analysis was performed as a case study in China [15, 16], with no *NPHS2* mutations reported in the Chinese CNS patients. The results of a single-gene analysis in Chinese SRNS patients showed that 1 out of 5 patients with CNS had a *WT1* mutation [18]. The number of studies reporting gene analyses of CNS patients is limited in China. Therefore, it is not clear which gene is the main cause of CNS in Chinese patients.

CNS is very rare in China. Mutations of *NPHS1*, *WT1* and *LAMB2* were identified in sporadic patients with CNS, and all of these investigations are case reports [15–17]. In this study, we performed analyses of *NPHS1*, *NPHS2*, *PLCE1*, *WT1*, *LAMB2*, *LMXIB*, and *COQ2* genes by direct sequencing in six Chinese children with CNS and whole exon sequencing (WES) in the other six children. The 12 children were from 12 unrelated families and 10 provinces and autonomous regions in China. Our results showed that eight patients had two heterozygous mutations in the *NPHS1* gene, one patient had a homozygous mutation in the *COQ6* gene, and one patient had a heterozygous mutation in the *WT1* gene. No mutations in any genes associated with nephrotic syndrome and proteinuria syndrome were detected in another two children by WES. The mutations identified by WES were all confirmed by Sanger sequencing. Mutations in the *NPHS1* and *COQ6* genes in patients were determined to arise from their parents by family analyses. Therefore, eight patients had compound heterozygous mutations in the *NPHS1* gene. This finding suggests that the *NPHS1* mutation is a major cause of CNS in Chinese patients. Our study only had 12 patients, but this group represented the largest cohort to date in China. An international cohort study in 2015, the largest cohort study performed thus far, revealed that a single-gene caused 69.4% of cases of SRNS that manifested in children in the first 3 months of life, and it revealed that the distribution of the causative genes was as follows: 40% for *NPHS1*, 10.6% for *NPHS2*, 8.5% for *WT1*, 5.5% for *LAMB2*, and 4.7% for all of the other genes combined [24]. Although there is a difference between our study and the international cohort study on the distribution of causative genes, *NPHS1* is a major causative gene of CNS in children in both studies.

CNS is a recessively inherited disorder that is characterised by a high degree of proteinuria at birth, a large placenta, and a marked oedema within the first 3 months of life [1, 25]. *NPHS1* was identified by positional cloning more than two decades ago. The Fin-major (c.121delCT; p.L41 fs) and Fin-minor (c.3325C > T; p.R1109X) mutations account for 78 and 16% of the mutated alleles, respectively, in Finnish cases; however, these mutations are rarely found in other ethnic groups [3, 4, 26]. In 2002, Koziell et al. reported the incidence of a Turkish CNS patient with a Fin-minor mutation [26]. To date, the Fin-minor mutation has only been reported in the populations of Finland and Turkey. Herein, we also documented the first occurrence of Fin-minor mutations outside of Finland and Turkey. Patient 5 had heterozygous IVS26DS-2A > T and Fin-minor mutations in intron and exon 26 of *NPHS1*, which arose from the patient’s father and mother, respectively. The IVS26DS-2A > T mutation was in the splicing junctions and was not found in the 100 control subjects in our study; furthermore, the mutation was in the highly conserved splice signals (‘AG’), and the prediction software programmes showed that it had a great effect on the splicing junctions (http://www.cbs.dtu.dk/services/NetGene2). Therefore, we deduced that the mutation affects the splicing process at the cDNA level. Patient 5 also had the typical symptoms of CNS, such as prematurity, a large placenta, a high degree of proteinuria, and rapid progression to ESRD (within 3 years). Two heterozygous nonsense mutations, Fin-minor and p.A1160X, were identified in patient 3. The p.R1160X mutation, a specific exon 27 nonsense mutation, is predicted to form a truncated protein that lacks the C-terminal 82 amino acids that are implicated in the interaction with podocin. However, patients with CNS caused by the p.R1160X mutation have an unexpectedly milder phenotype, and most of these individuals are females, suggesting a gender effect [27]. Surprisingly, all affected patients are, reportedly, homozygous for this mutation and, among those in whom a renal biopsy is performed, the histologic findings are consistent with CNS [27]. Nevertheless, these patients either have a mild degree of proteinuria or go into remission between the ages of 5- and 19-years-old [27]. Patient 3 had serious features of nephrotic syndrome, and no improving trend of the disease during the follow-up period (3 months). He might have had a poor prognosis because of his heterozygous p.R1160X and Fin-minor mutations, which is different from patients with homozygous p.R1160X mutations. Our study showed that a Fin-minor mutation was detected in two of the eight CNS patients (25%) with a *NPHS1* mutation. Although our patients were different from those in the Finnish study on race[], a Fin-minor mutation is also a common mutation in our patients.

Of these 12 children, one child (case 12) with extrarenal symptoms had a homozygous c.1078C > T (p.R360W) mutation in the *COQ6* gene. The homozygous p.R360W mutation has been not reported before and is damaging for the COQ6 protein, as predicted by the PolyPhen and SIFT software. The COQ6 protein was expressed almost exclusively in the glomeruli, rather than in the tubules [28]. The *COQ6* gene mutations that cause a CoQ10 deficiency led to the nephrotic syndrome [28]. The child had been treated with CoQ10 since his genetic diagnosis when he was one year old. His proteinuria was observed to be in complete remission at three months after treatment with 30 mg/ (kg.d) CoQ10. It was first instance of a reported mutation in the *COQ6* gene in Chinese children with CNS. A heterozygous c.1334C > T mutation in the *WT1*gene was detected in case 11 by WES, which was confirmed by Sanger sequencing. Sanger sequencing in the family of case 12 showed that the c.1334C > T (p.R445Q) mutation was de novo, which had been not reported beforehand. Prediction by the PolyPhen and SIFT software displayed the c.1334C > T mutation as being damaging for the WT1 protein. Case 11 progressed to end-stage renal disease shortly after the diagnosis of CNS, and no incidences of sex differentiation disorder, Wilms tumour, and gonadoblastoma was found in this patient to date. Patients with the *WT1* mutation not only more frequently presented with chronic kidney disease and hypertension at the time of diagnosis and exhibited a more rapid disease progression but also more frequently had incidences of sex reversal and/or urogenital abnormalities, Wilms tumour, and gonadoblastoma [29]. Therefore, more attention should be paid to this patient, in order to find an occurrence of Wilms tumour and gonadoblastoma in a timely fashion.

Based on our data, which showed that the *NPHS1* mutation is the major cause of CNS in Chinese patients, we recommend a *NPHS1* analysis in Chinese patients with CNS. Because NPHS1 mutations are distributed among different exons and splicing mutations, a mutational analysis should be performed in all NPHS1 exons, as well as at the boundaries of the introns and exons. However, mutations in several genes can cause CNS. With a decline in sequencing costs, next generation sequencing is an optimal choice for gene screening in patients with CNS, with next generation sequencing including disease targeted sequencing and WES.

## Conclusions

Our study demonstrates that the majority of CNS cases are caused by genetic defects, and the *NPHS1* mutation is the most common cause of CNS in Chinese patients. A mutational analysis of *NPHS1* should be recommended in Chinese patients with CNS in all exons of *NPHS1* and in the intron-exon boundaries.

## Abbreviations

CNS: Congenital nephrotic syndrome
ESRD: End-stage renal disease
HIV: Human immunodeficiency virus
MAF: Minor allele frequency
PCR: Polymerase chain reactions
WES: Whole exome sequencing
